# Mitochondrial 3243A > G mutation confers pro-atherogenic and pro-inflammatory properties in MELAS iPS derived endothelial cells

**DOI:** 10.1038/s41419-019-2036-9

**Published:** 2019-10-22

**Authors:** Nicole Min Qian Pek, Qian Hua Phua, Beatrice Xuan Ho, Jeremy Kah Sheng Pang, Jin-Hui Hor, Omer An, Henry He Yang, Yang Yu, Yong Fan, Shi-Yan Ng, Boon-Seng Soh

**Affiliations:** 1 Disease Modeling and Therapeutics Laboratory, A*STAR Institute of Molecular and Cell Biology, 61 Biopolis Drive Proteos, Singapore, 138673 Singapore; 2 Department of Biological Sciences, National University of Singapore, Singapore, 117543 Singapore; 3 Neurotherapeutics Laboratory, A*STAR Institute of Molecular and Cell Biology, 61 Biopolis Drive Proteos, Singapore, 138673 Singapore; 4 Cancer Science Institute of Singapore, National University of Singapore, Singapore, 117599 Singapore; 5 Center of Reproductive Medicine, Department of Obstetrics and Gynecology, Peking University Third Hospital, Beijing, 100191 China; 6 Key Laboratory for Major Obstetric Diseases of Guangdong Province, The Third Affiliated Hospital of Guangzhou Medical University, Guangzhou, 510150 China; 7 National Neuroscience Institute, 11 Jalan Tan Tock Seng, Singapore, 308433 Singapore; 8 Department of Physiology, National University of Singapore, 2 Medical Dr, Singapore, 117593 Singapore

**Keywords:** Induced pluripotent stem cells, Atherosclerosis, Stroke, Chronic inflammation

## Abstract

Mitochondrial encephalomyopathy, lactic acidosis, and stroke-like episodes (MELAS) syndrome is a mitochondrial disorder that is commonly caused by the m.3243A > G mutation in the *MT-TL1* gene encoding for mitochondrial tRNA(Leu(UUR)). While clinical studies reported cerebral infarcts, atherosclerotic lesions, and altered vasculature and stroke-like episodes (SLE) in MELAS patients, it remains unclear how this mutation causes the onset and subsequent progression of the disease. Here, we report that in addition to endothelial dysfunction, diseased endothelial cells (ECs) were found to be pro-atherogenic and pro-inflammation due to high levels of ROS and Ox-LDLs, and high basal expressions of VCAM-1, in particular isoform b, respectively. Consistently, more monocytes were found to adhere to MELAS ECs as compared to the isogenic control, suggesting the presence of an atherosclerosis-like pathology in MELAS. Notably, these disease phenotypes in endothelial cells can be effectively reversed by anti-oxidant treatment suggesting that the lowering of ROS is critical for treating patients with MELAS syndrome.

## Introduction

Mitochondrial encephalomyopathy, lactic acidosis and stroke-like episodes (MELAS) syndrome is one of the most common maternally inherited mitochondrial disorders with 80% of MELAS patients carrying the m.3243A > G mutation in the *MT-TL1* gene that encodes for tRNA (Leu-UUR)^1,2^. Transfer RNA (Leu-UUR) plays critical roles in the translation of proteins essential for the assembly and function of mitochondrial complexes in the electron transport chain. As such, the translational defects caused by the m.3243A > G gene mutation disrupts the oxidative phosphorylation function of the electron transport chain. Defective energy production cause energetic stress particularly in cells with high energetic demands such as neurons and myocytes leading to a myriad of secondary consequences. The most direct consequence of the m.3243A > G mutation in *MT-TL1* gene is complex I (CI) deficiency^3^, which results in accumulation of NADH, the main substrate of CI, which then accelerate rate of glycolysis in order to compensate for the reduced ATP production. Lactate, an end-product of glycolysis, eventually accumulates and leads to lactic acidosis that is observed in MELAS patients^4^. Resultant inefficient energy production to cope with energetic stress has also been associated with oxidative stresses found in MELAS^5^.

The severity of MELAS is dependent on the heteroplasmy level of mutant DNA in cells of an individual^6,7^. Stroke-like episodes are the most debilitating symptom of MELAS^8^, and it manifest itself similarly to ischaemic stroke but differs in the characteristics of the brain lesions and accompanying symptoms such as visual impairment, blurred vision and dementia^9^. At present, while there are evidences documenting the presence of atherosclerotic lesions in aortic tissues of MELAS patients^10–12^, it remained unclear how m.3243A > G mutation presents a focal point for atherogenesis at the vasculature of MELAS patients, thereby increasing their susceptibility to atherosclerosis.

In this study, we sought to understand the mechanisms underlying the pathogenesis of SLEs in MELAS by utilising induced pluripotent stem cells (iPSCs) derived from MELAS patient harbouring a high proportion (>80%) of m.3243A > G mutant mtDNA^13^. The diseased ECs present distinct mitochondria abnormalities that were reported in vessels of MELAS patients. Both molecular and functional assays performed on the ECs revealed endothelial dysfunction in MELAS ECs with pro-atherogenic and pro-inflammation properties. These MELAS ECs were also found to express high levels of VCAM-1 isoform b, which is thought to be indicative of the ‘activated’ status of ECs^14^. The diseased phenotypes, however, were alleviated with anti-oxidant treatment, suggesting that reactive oxygen species (ROS) play crucial role in MELAS disease onset and progression.

## Results

### MELAS iPSCs have reduced efficiency to differentiate into ECs

**Fig. 1:**
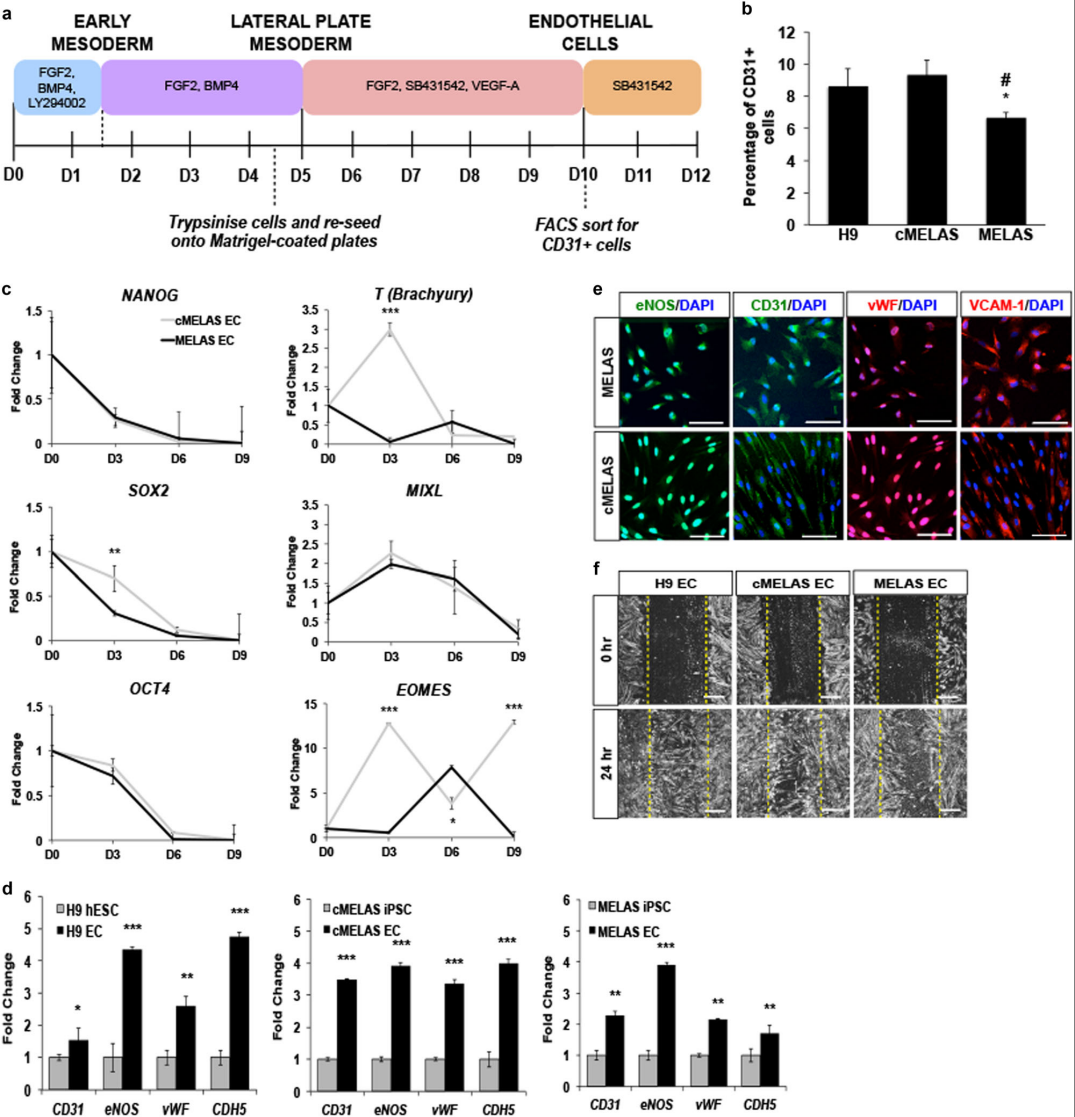
MELAS iPSCs have reduced efficiency to differentiate into ECs. **a** Schematic diagram illustrating the differentiation protocol utilised to generate ECs from hPSCs. On day 10, cells expressing CD31 would be isolated for further expansion. **b** Flow cytometry analysis of CD31^+^ ECs. There were lower percentage of CD31^+^ ECs in cells differentiated from MELAS iPSCs as compared to WT and isogenic control. **c** Time-course expression analysis of pluripotent genes (*NANOG, SOX2* and *OCT4*) and mesodermal genes (*T, MIXL* and *EOMES*) showed lower expression of *T* and *EOMES* at day 3 of MELAS EC differentiation. **d** Expression of specific EC gene markers *CD31*, *eNOS, vWF* and *CDH5* in CD31^+^ cells were significantly higher than the respective hPSCs. Data are represented as fold-change normalised to *β-ACTIN*. **e** Representative images of eNOS, CD31, vWF and VCAM-1 staining in cMELAS and MELAS ECs. Nuclei were stained in blue with DAPI. Scale bar = 100 μm. **f** Scratch assay performed using ECs derived from H9, cMELAS and MELAS iPSCs. The ability to migrate to the scratch area after 24 h showed the functionality in these ECs. Error bars show SD of the mean. *p < 0.05, **p < 0.01, ***p < 0.001

Endothelial cells were derived from three hPSC cell lines, namely H9, MELAS and cMELAS iPSCs using an established protocol^15^ (Fig. 1a, S1a). Purified CD31 + ECs were then cultured in EGM-2 supplemented with SB431542 and left to recover and proliferate for 2–3 days. During the course of differentiation, more cell death was observed amongst cells that were differentiating from MELAS iPSCs (Day 5) (Fig. S1b). In addition, there were significantly fewer CD31 + cells obtained from MELAS iPSC (6.67%) as compared to H9 (8.61%) and the isogenic control (9.34%) (Fig. 1b), indicating that MELAS iPSCs differentiate less efficiently into CD31 + ECs.

Additionally, quantitative PCR analysis of pluripotency and mesodermal genes at days 0, 3, 6 and 9 revealed that MELAS iPSCs have significantly reduced expression of mesodermal genes *T* (*Brachyury)* and *EOMES* at day 3 of differentiation when compared to cMELAS iPSCs, a time point where cells are differentiating towards the mesoderm lineage (Fig. 1c). Confirming that MELAS iPSCs were not refractory to differentiation, NANOG, SOX2 and OCT4 were similarly downregulated in MELAS and cMELAS cultures, even though MELAS cultures showed a greater extend of SOX2 downregulation at day 3 (Fig. 1c, S1c). Although EC differentiation from MELAS iPSCs was less efficient, the ECs derived from all three hPSC lines were shown to express specific endothelial markers such as CD31, endothelial nitric oxide synthase (eNOS), von-Willebrand Factor (vWF), Ve-Cadherin (VeCAD) (encoded by *CDH5* gene) and vascular cell adhesion molecule-1 (VCAM-1), both at the transcript as well as at the protein levels (Fig. 1d, e). Functionally, ECs derived from all the three hPSC lines demonstrated cellular migration capabilities as shown in the scratch assay (Fig. 1f).

### Increased mitochondrial biogenesis and oxidative stress detected in MELAS ECs

**Fig. 2:**
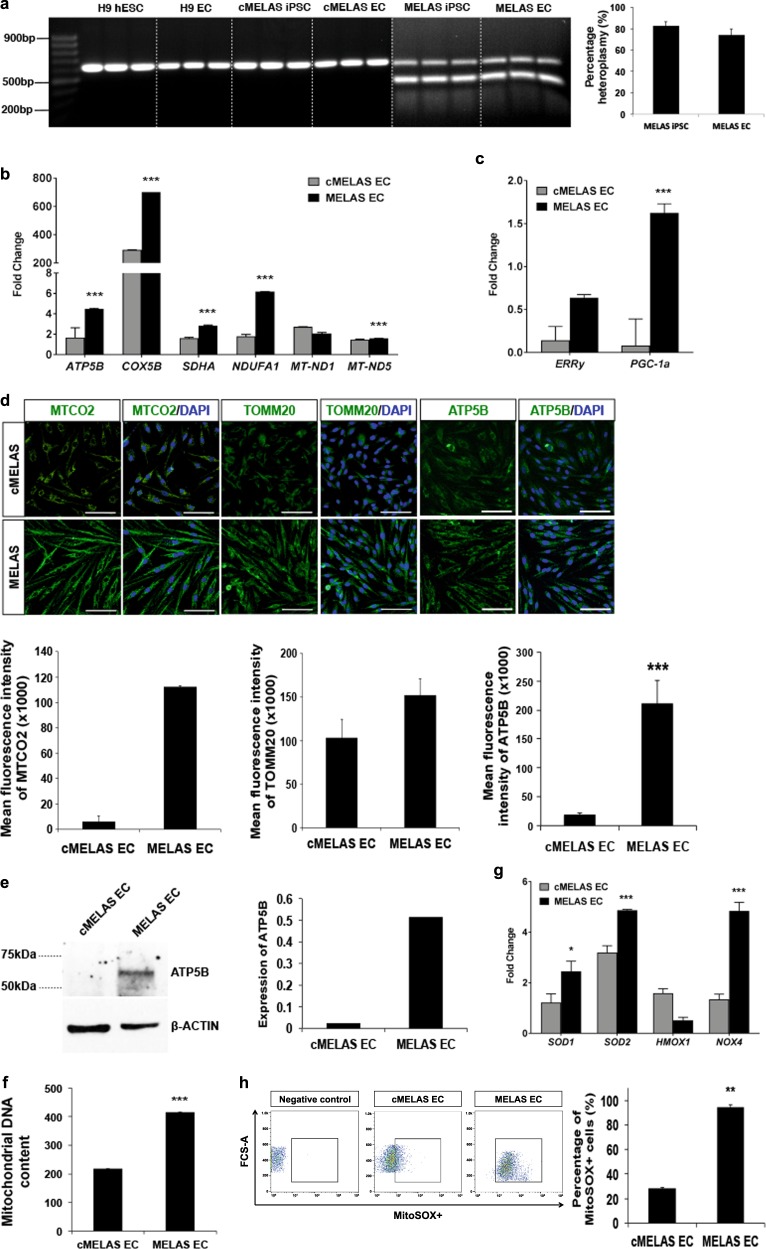
MELAS ECs recapitulate mitochondrial aberrations associated with m.3243 A > G mutation. **a** RFLP analysis of the m.3243 locus of the *MT-TL1* genes in MELAS iPSC and MELAS ECs showed high levels of m.3243A> G heteroplasmy while m.3243A > G mutant mtDNAs were absent in the WT and isogenic controls. Percentage of heteroplasmy between MELAS iPSC and MELAS ECs was non-significant. **b** Quantitative-PCR analysis demonstrates up-regulation of mitochondria OX-PHOS genes in MELAS ECs. Data are represented as fold-change normalised to *β-ACTIN*. **c** Quantitative-PCR analysis demonstrates up-regulation of mitochondria biogenesis genes in MELAS ECs. Data are represented as fold-change normalised to *β-ACTIN*. **d** Representative images of MTCO2, ATP5B and TOMM20 immunostaining in cMELAS and MELAS ECs. Nuclei were stained in blue with DAPI. Scale bar = 100 μm. The graph shows increased mean fluorescence intensity of MTCO2, ATP5B and TOMM20 in MELAS EC. **e** Western blot and densitometric analysis shows higher ATP5B protein expression in MELAS ECs as compared to cMELAS ECs. **f** Mitochondrial DNA content which is determined by normalising mtDNA to nuclear DNA copy number was found to be significantly higher in MELAS ECs as compared to the control. **g** Quantitative-PCR analysis demonstrates up-regulation of oxidative stress-related genes in MELAS ECs. Data are represented as fold-change normalised to *β-ACTIN*. **h** Mitochondrial superoxide production was detected with MitoSOX with FACS sorting. Flow analysis demonstrates higher percentage of MELAS ECs cells producing mitochondrial superoxide as compared to cMELAS ECs. Error bars show SD of the mean. **p < 0.01, ***p < 0.001. n.s., not significant

To verify that mutant mtDNA heteroplasmy levels were unchanged during EC differentiation, restriction fragment length polymorphism (RFLP) analysis was performed. The results indicated MELAS iPSCs and resultant ECs have a high heteroplasmy level of ~80% (Fig. 2a). As clinical manifestations of mitochondrial disorders are typically caused by dysregulations of energy production, we examined the expression levels of several genes encoding for various mitochondrial complexes in the electron transport chain (ETC) such as ATP synthase subunit beta (*ATP5B*), cytochrome c oxidase subunit 5B (*COX5B*), succinate dehydrogenase complex subunit A (*SDHA*), NADH:ubiquinone oxidoreductase subunit A1 (*NDUFA1*), NADH-ubiquinone oxidoreductase chain 1 and 5 (*MT-ND1*and *MT-ND5)*. At the mRNA level, MELAS ECs showed increased expressions of *ATP5B*, *COX5B*, *SDHA* and *NDUFA1*, as well as *PGC1α* and *ERRγ*, key genes involved in mitochondrial biogenesis (Fig. 2b, c). Further validation via immunostaining of ECs showed increased expression of cytochrome c oxidase subunit 2 (MTCO2), mitochondrial import receptor subunit TOM20 homologue (TOMM20) and ATP5B in MELAS ECs (Fig. 2d). Live staining of mitochondria in MELAS ECs using Mitotracker also revealed a 2-fold increase in mean fluorescence intensity, significantly higher than the control ECs (*p* < 0.001) (Fig. S2a). Consistently, MELAS ECs were also found to express more ATP5B protein (Fig. 2e), and qPCR-based determination of mtDNA content further revealed that diseased ECs have significantly higher level of mtDNA content when compared to control ECs (*p* < 0.001) (Fig. 2f), confirming that MELAS ECs have a higher mitochondrial biogenesis rate than controls.

Being the energy powerhouse of the cell, mitochondria are one of the main sources of reactive oxygen species (ROS), and MELAS has been previously reported to be associated with increased oxidative stress due to defects of the electron transport chain^5,16^. To determine if MELAS iPSC-derived ECs were indeed producing more ROS as a result of increased production and accumulation of mitochondria, we examined the expressions of oxidative stress related genes, as well as mitochondrial superoxide level in diseased ECs. Indeed, increased mRNA levels of *SOD1*, *SOD2* and *NOX4* were observed (Fig. 2g). Significant increase of mitochondrial superoxide in the diseased ECs (~3-fold; *p* < 0.001) was revealed by the MitoSOX assay (Fig. 2h). Taken together, we confirm a unique mitochondrial aberration in MELAS ECs characterised by increased mitochondrial biogenesis as well as increased mitochondrial reactive oxygen species.

### MELAS ECs are pro-apoptotic and exhibit tube formation defects

**Fig. 3:**
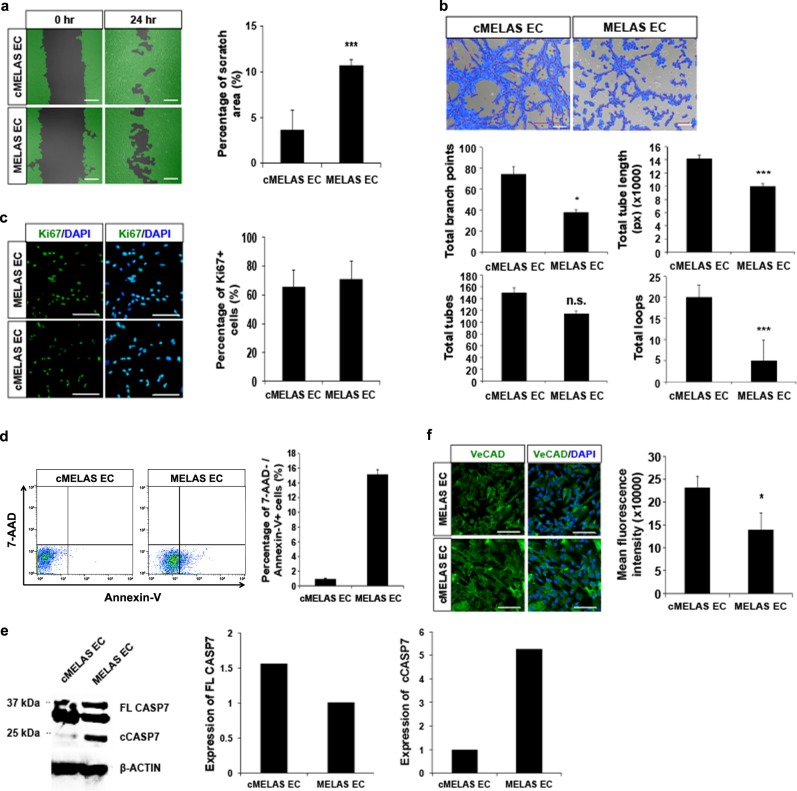
MELAS ECs exhibit functional defects. **a** Scratch wound healing assays was performed on cMELAS and MELAS ECs. After 24 h, scratch area of MELAS ECs was observed to remained larger than the control, suggesting inefficient migration in MELAS ECs. Graphical representation illustrates the percentage of scratch area left after 24 h. **b** Tube formation assay was performed on cMELAS and MELAS ECs. Representative images demonstrate reduced tube formation in MELAS ECs. Quantitative analysis of several tube formation parameters showed overall poorer tube formation capacity of MELAS ECs. **c** Representative images of Ki67 immunostaining in cMELAS and MELAS ECs. Nuclei were stained in blue with DAPI. Scale bar = 100 μm. No distinct changes in percentage of Ki67 + cells between control and MELAS ECs (*n* > 100). **d** Flow analysis of Annexin-V showed higher percentage of MELAS ECs that were undergoing apoptosis. **e** Western blot and densitometric analysis shows higher levels of cleaved CASP7 protein expression in MELAS ECs as compared to cMELAS ECs. Expression of cleaved CASP7 was quantified and normalised to loading control β-ACTIN. **f** Representative images of cMELAS and MELAS ECs stained with VeCAD. Nuclei were stained in blue with DAPI. Scale bar = 100 μm. Quantification of mean fluorescence intensity illustrates MELAS ECs expressed lower levels of VeCAD protein. Error bars show SD of the mean. *p < 0.05, ***p < 0.001

With the innate mitochondrial defects, we postulate that MELAS ECs would possess functional deficits. In this regard, we performed cellular migration and tube formation assays in MELAS and cMELAS ECs. In the scratch assay, quantification of the extent of migration revealed that the scratch area 24 h post-scratch of MELAS ECs remains larger than that of the isogenic control ECs (>1-fold) (Fig. 3a). In addition, tube formation assay demonstrated that MELAS ECs performed significantly poorer in several tube formation parameters – decrease in total tube length by 30%, decrease in total branch point by 49%, 75% reduction in total loops and 24% reduction in total tubes (Fig. 3b).

To examine the contributing factors underlying these functional defects observed in MELAS ECs, cellular processes such as proliferation and apoptosis were investigated. While there are no significant differences in the number of Ki67 + cells between ECs derived from either MELAS or cMELAS iPSCs (Fig. 3c), a greater proportion of diseased ECs (~15%) were found to be apoptotic (7-AAD- Annexin-V+), compared to controls (~1%) (Fig. 3d). This pro-apoptotic profile of MELAS ECs was confirmed by western blot using antibodies against Caspase-7, revealing increased cleaved CASP7 (cCASP7) in MELAS ECs (Fig. 3e).

As cell-to-cell adherence and communication is essential for angiogenesis, the inability of MELAS ECs, then, to form proper tubes in vitro could be a consequence of alterations to levels of adherent proteins. As such, to account for the poor tube formation capabilities in MELAS, we examined the expression of VeCAD, a key adherent junction protein for angiogenesis^17,18^. Quantitative immunofluorescence revealed that MELAS ECs indeed express significantly less VeCAD (Fig. 3f). Collectively, the pro-apoptotic status and downregulated VeCAD expression could provide an explanation for reduced tube formation observed in MELAS ECs.

### MELAS ECs display dyslipidemia and LDL-induced inflammatory responses

**Fig. 4:**
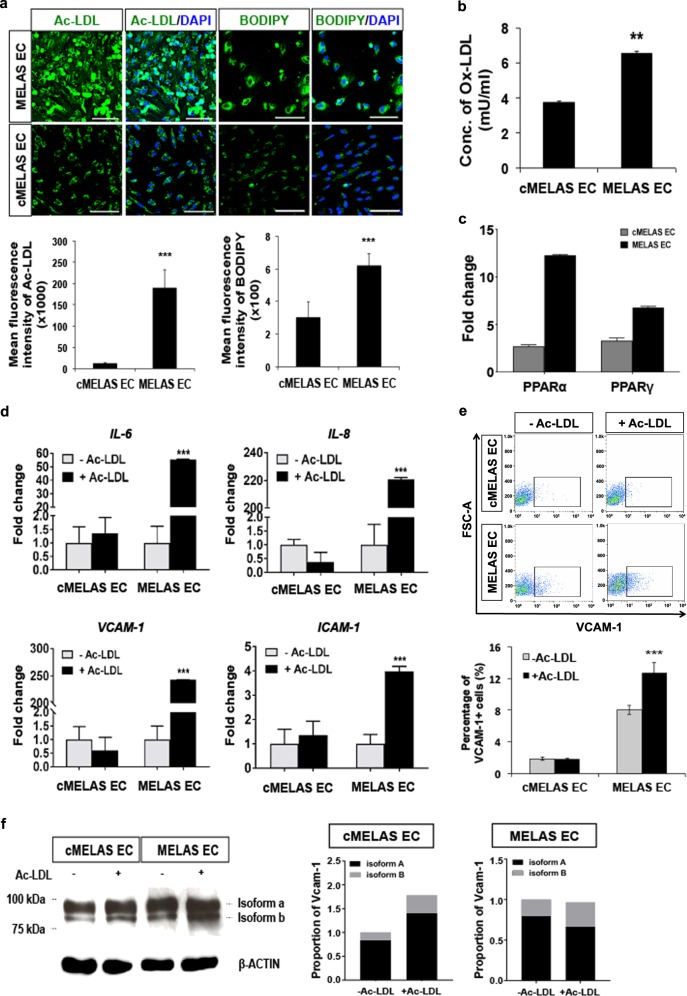
Evidence of dyslipidemia and LDL-induced inflammatory responses in MELAS ECs. **a** Representative images of Ac-LDL and BODIPY staining in cMELAS and MELAS ECs. Nuclei were stained in blue with DAPI. Scale bar = 100 μm. Quantification of mean fluorescence from *n* > 100 cells illustrated an increase in Ac-LDL and neutral lipids in MELAS ECs as compared to cMELAS ECs. **b** ELISA analysis showed higher level of ox-LDL detected in cell lysate of MELAS ECs. **c** Quantitative-PCR analysis illustrated increased mRNA transcripts expression of both *PPARα* and *PPARγ* in MELAS ECs as compared to the isogenic control. Data are represented as fold-change normalised to *β-ACTIN*. **d** Quantitative-PCR analysis demonstrated up-regulation of inflammatory markers *ICAM-1, VCAM-1, IL-8* and *IL-6* in MELAS ECs upon Ac-LDL treatment. Data are represented as fold-change normalised to *β-ACTIN*. **e** Flow cytometric analysis of VCAM-1 showed that Ac-LDL treatment resulted in more VCAM-1 + cells in MELAS ECs than the control. **f** Western blot and densitometric analysis showed higher levels of VCAM-1 isoform b protein expression in MELAS ECs as compared to cMELAS ECs after Ac-LDL treatment. Expression of VCAM-1 isoforms was quantified and normalised to loading control β-ACTIN. Error bars show SD of the mean. **p < 0.01, ***p < 0.001

One of the key roles of ECs in cholesterol metabolism is the ability to uptake modified low-density lipoprotein (LDL) in circulation^19^. To investigate if MELAS ECs have a differential ability to take up modified LDL, we incubated Alexa-Fluor 488-conjugated Ac-LDL with ECs derived from MELAS and cMELAS iPSCs, and observed significantly higher Ac-LDL uptake by MELAS ECs (*p* < 0.001) (Fig. 4a). To confirm this finding, EC cells were also stained for BODIPY 493/503, a neutral lipid stain, which demonstrated that neutral lipid content is indeed higher in MELAS ECs (Fig. 4a).

It was earlier demonstrated that MELAS ECs possess significantly higher levels of oxidative stress (Fig. 2g, h). Expanding on our findings, we sought to investigate if this increased LDL uptake could explain the stroke-like episodes that MELAS patients experience. To this end, we analysed MELAS and cMELAS cell lysates for oxidised-LDL (ox-LDL) content, as it is well-established that pathologically high levels of ox-LDL accumulated in the endothelium can promote atherogenesis^20^. Ox-LDL enzyme-linked immunosorbent assay (ELISA) confirmed that MELAS ECs contained 0.5-fold higher concentration of ox-LDL (~6 mU/ml) as compared to control (Fig. 4b). Consistently, *PPARα* and *PPARγ*, downstream effectors of ox-LDL, were upregulated in MELAS ECs (*p* < 0.001) (Fig. 4c), confirming that an elevated level of ox-LDL is capable of promoting the activation of downstream signalling pathways^21^.

In addition, examining the expressions of several LDL receptor genes such as lectin-like oxLDL receptor-1 (*LOX-1*), LDL receptor (*LDLR*), fatty-acid translocase (*CD36*) in MELAS ECs revealed no significant differences in their mRNA expressions as compared to the isogenic control. Instead, the expression of low-density lipoprotein receptor-related protein 1 (*LRP1*), a receptor involved in receptor-mediated endocytosis, was found to be highly upregulated in MELAS ECs (Figure S3a). Interestingly, lipoprotein lipase (LPL) was significantly downregulated in MELAS ECs (Figure S3a), suggesting that the observed increase in Ac-LDL and BODIPY staining in MELAS ECs could be due to a combination of heightened endocytic uptake and reduced degradation of modified LDLs in the diseased ECs. Taken together, this suggests that high endogenous levels of ox-LDL could be a result of increased ROS in MELAS ECs (Fig. 2h).

As inflammation and artherosclerosis are intricately linked, we examined the expression levels of inflammatory genes such as *ICAM-1, VCAM-1, IL-8 and IL-6* upon exposure to Ac-LDL, and confirmed the up-regulation of all these genes in MELAS ECs compared to its isogenic control (*p* < 0.001) (Fig. 4d). Consistently, a significantly higher proportion of MELAS ECs were found to express VCAM-1 (Fig. 4e), which is a cytokine-inducible surface molecule that promotes adhesion of immune cell types, and alternative splicing can give rise to a few isoforms of VCAM1^22,23^. Notably, we found that the smaller VCAM-1 isoform b was significantly elevated in MELAS ECs in response to Ac-LDL (Fig. 4f, S4a). This abnormal inflammatory response to Ac-LDL exhibited by the MELAS ECs is unlikely to be due to lipotoxicity, as described in other studies^24,25^, as exposure to Ac-LDL did not result in an increase in cell death (Figure S3b). It is instead likely to be due to the high levels of basal ROS in MELAS ECs as treating MELAS ECs with 300 μM of Vitamin C (Vit C), a known antioxidant, reduced Ac-LDL fluorescence intensity in MELAS ECs (*p* < 0.05) by ~20% (Fig. S3c).

### MELAS ECs display dysregulated inflammatory responses

**Fig. 5:**
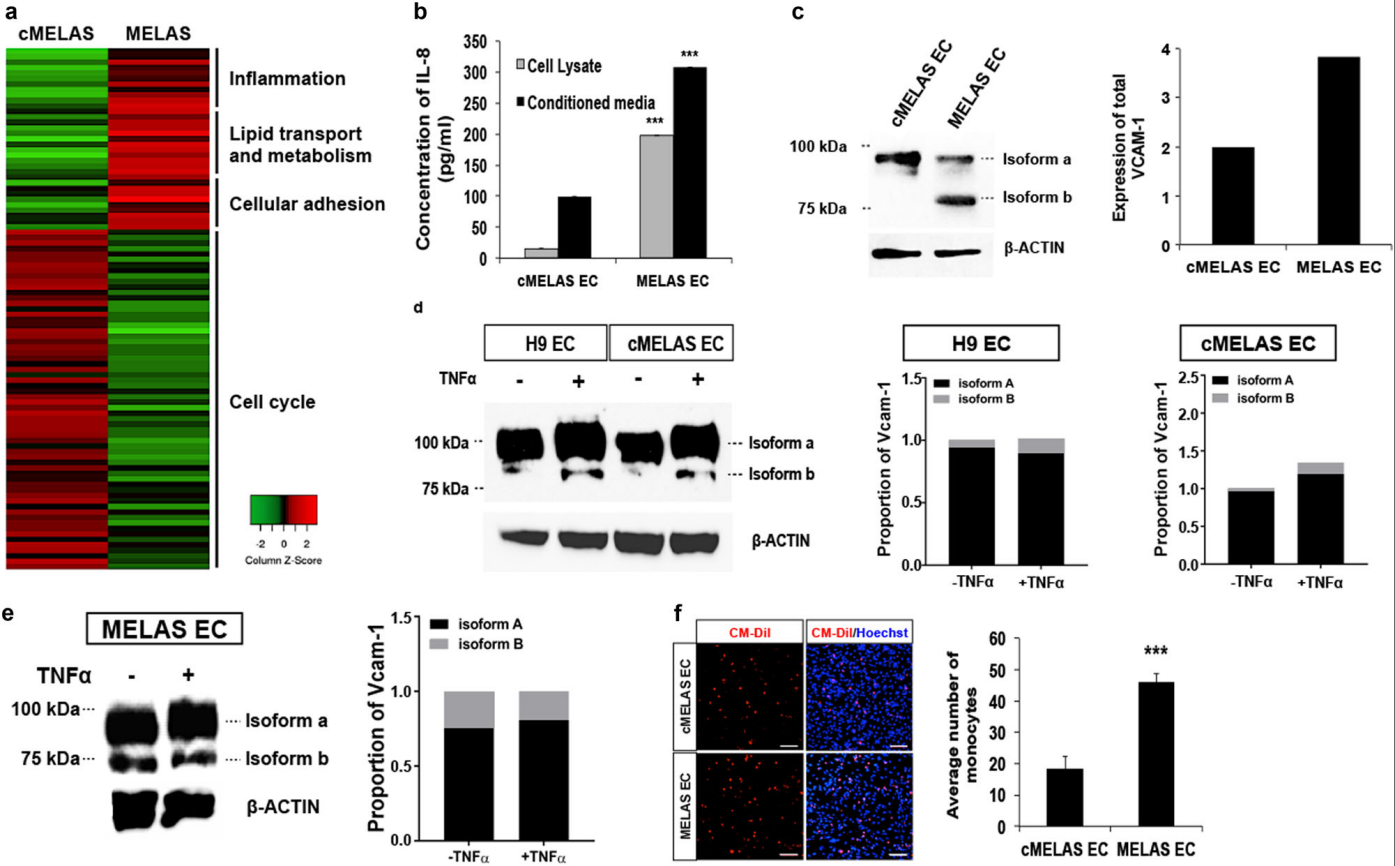
Dysregulated inflammatory responses were observed in MELAS ECs. **a** Gene ontology enrichment analysis revealed gene clusters that are differentially expressed between MELAS ECs and cMELAS ECs. **b** ELISA analysis demonstrated higher levels of IL-8 detected in the conditioned media and cell lysate of MELAS ECs. **c** Western blot and densitometric analysis showed presence of VCAM-1 isoform b protein expression in MELAS ECs as compared to cMELAS ECs. Expression of total VCAM-1 were quantified and normalised to loading control β-ACTIN. **d** Western blot and densitometric analysis showed elevated levels of VCAM-1 isoform b expressions in control ECs upon TNFα treatment. Expression of VCAM-1 isoforms were quantified and normalised to loading control β-ACTIN. **e** Western blot and densitometric analysis showed no further increase in VCAM-1 isoform b expression level when MELAS ECs were treated with TNFα. **f** Monocyte adhesion assay was performed and demonstrated significantly more monocytes attached to MELAS ECs as compared to the isogenic control. Nuclei were stained in blue with DAPI. Scale bar = 100 μm. Error bars show SD of the mean. ***p < 0.001

In order to unravel key mechanistic differences between diseased and control ECs, RNA-Seq was performed on cMELAS and MELAS ECs. Gene ontology enrichment analysis revealed up-regulation of gene clusters related to inflammation, lipid transport and metabolism and cellular adhesion in MELAS ECs and also downregulation of cell cycle genes (Fig. 5a). A comprehensive list of differentially expressed genes analysed is provided in Supplementary Tables 1 and 2.

To validate if MELAS ECs do have an intrinsically heightened level of inflammation, ELISA assay on IL-8 was performed on cell lysates and conditioned media harvested from both diseased and control ECs. The results revealed that MELAS ECs expressed significantly higher levels of IL-8 in both cell lysate and conditioned media at 10-folds and 3-folds higher, respectively (Fig. 5b). As VCAM-1 is a crucial adhesion molecule expressed by ECs during inflammation, and plays key role in leucocyte-endothelial cell interaction before leucocytes transmigrate through vascular walls^26^, we compared its expressions in both MELAS and cMELAS ECs. Expectedly, total VCAM-1 protein expression was found to be higher in MELAS ECs (Fig. 5c, S4a), with the diseased ECs specifically expressing a second isoform (herein coined as isoform b), which is distinctively absent in the control ECs (Fig. 5c).

At present, not much is known about VCAM-1 isoforms but at a structural level, they are reported to differ at the extracellular domain (Fig. S4b). VCAM-1 isoform b is thought to be the ‘pro-adhesive’ isoform of VCAM-1 and is indicative of the ‘activation’ status of ECs^14^. Physiologically, ECs are activated in response to stresses and are known to be both pro-inflammatory and pro-thrombotic^27^. This prompted us to investigate if VCAM-1 isoform b would be differentially expressed when MELAS ECs and control ECs are treated with tumour necrosis factor-α (TNFα) or tunicamycin, an inflammatory and ER stress inducer, respectively. As shown in Fig. 5d, S4c, upon TNFα or tunicamycin treatment for 48 h, wild-type ECs (derived from H9 and isogenic control) were demonstrated to express this ‘pro-adhesive’ VCAM-1 isoform b at significantly higher levels. In contrast, VCAM-1 isoform b expression did not increase in MELAS ECs upon TNFα treatment, suggesting that VCAM-1 isoform b could already be maximally expressed in MELAS ECs (Fig. 5e).

To confirm that MELAS ECs display a heightened level of basal inflammation, immunocytochemistry of NF-_Κ_B p65 (RelA) upon TNFα stimuli was performed and the results revealed higher levels of NF-_Κ_B nuclear localisation in MELAS ECs as compared to controls (Fig. S4d). Consistently, mRNA expression of NF-_Κ_B was upregulated in MELAS ECs upon TNFα stimulation (Fig. S4e). To further examine basal and stimulated (TNFα treated) inflammation levels in MELAS ECs, we investigated the expression of NLRP3, a NOD-like receptor protein that recruits the inflammasome-adaptor protein ASC (PYCARD), which interacts with CASP-1 leading to its activation that promotes the maturation of proinflammatory cytokine, IL-1β. The results observed in Figure S4f indicated heightened basal expressions of inflammasome components including *NLRP3*, *ASC* and *CASP1*, as well as *IL-1β* and adhesion molecules (*ICAM-1* and *VCAM-1*). Protein levels of some of these expressed genes such as ASC, CASP1 and activated CASP1 (Fig. S4g) further validated heightened inflammation observed in MELAS ECs under both basal and stimulated conditions. Taken together, this indicates that MELAS ECs are chronically activated; experiencing a heightened level of inflammation.

In order to draw a direct correlation between the chronically activated status of MELAS ECs (expression of VCAM-1 isoform b) to the pathogenesis of atherosclerosis, we compared the adhesion ability of monocytes to ECs derived from MELAS iPSC or the isogenic control. With higher expression of ‘pro-adhesive’ VCAM-1 isoform b in MELAS ECs, it was observed that at a basal level (unstimulated condition), significantly more monocytes (>2-fold) adhered to the diseased ECs (Fig. 5f), indicating that MELAS ECs are highly adhesive and are thus, primed for inflammation.

### Anti-oxidant treatments demonstrated to improve endothelial functions in MELAS ECs

**Fig. 6:**
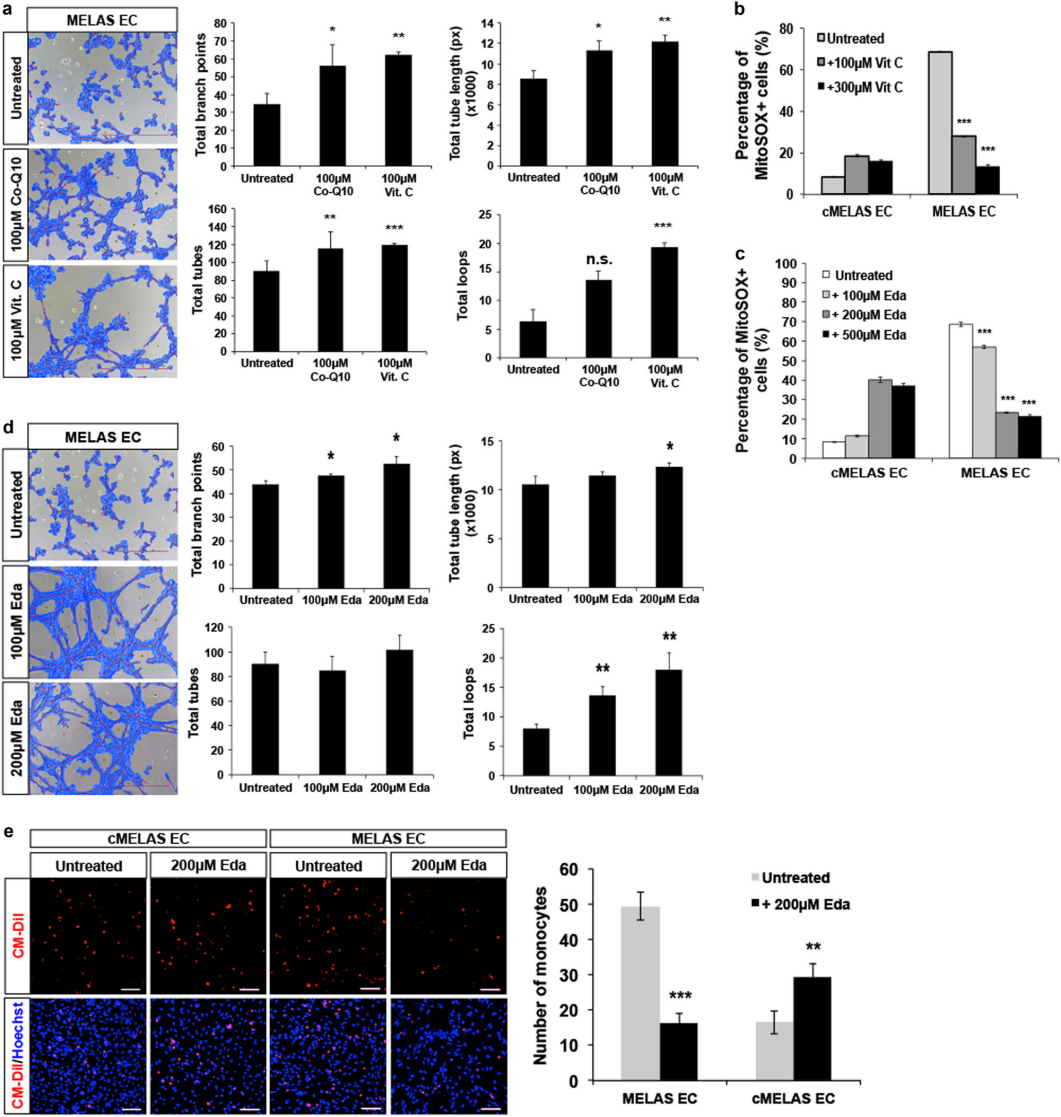
Anti-oxidant treatments were successful in improving endothelial functions. **a** Representative images of tubes formed by MELAS ECs pre-treated with 100 μM Vit. C and 100 μM Co-Q10. Quantitative analysis of several tube formation parameters showed improvements in tube formation capacity after MELAS ECs were treated with the compounds. **b** 100 μM Vit. C treatment was effective in reducing the number of MitoSOX^+^ cells in MELAS ECs. **c** Increasing doses of edaravone was effective in reducing MitoSOX^+^ cells in MELAS ECs in a dose-dependent manner. **d** Representative images of tubes formed by MELAS ECs treated with 100 μM and 200 μM of edaravone. Quantitative analysis of several tube formation parameters showed improvements in tube formation capacity after MELAS ECs were treated with both doses of edaravone. **e** MELAS ECs treated with 200 μM of edaravone effectively lowered number of monocytes adhering to MELAS ECs when monocytes adhesion assay was performed. Nuclei were stained in blue with DAPI. Scale bar = 100 μm. Error bars show SD of the mean. *p < 0.05, **p < 0.01, ***p < 0.001

We envisage that the pathologically high levels of oxidative stress underpin endothelial dysfunction, and in part, the pro-atherogenic property observed in MELAS ECs. Therefore, anti-oxidant treatments targeted at lowering levels of ROS in MELAS ECs was investigated to determine if the defects could be improved or reversed. As co-enzyme Q10 (co-Q10) was previously reported to be therapeutic for the treatment of cardiovascular events^28^ and mitochondrial disorders including MELAS^29,30^, it was included as a positive control in our assays. Utilising co-Q10 (100 μM) and Vit. C (100 μM) to treat MELAS ECs, several tube formation parameters were assessed and compared (Fig. 6a). The results demonstrated that co-Q10 is notably not as efficient in restoring tube formation capability in MELAS ECs as compared to Vit C as increasing doses of Vit C was able to lower mitochondrial superoxide levels in MELAS ECs (Fig. 6b, S5a). Consistently, increasing doses of Vit C was also observed to effectively reduce expression of inflammasome components such as ASC and CASP1 (Fig. S5b).

In this regard, we further tested the efficacy of a recently FDA approved drug, edaravone (Eda) which is a potent anti-oxidant. Increasing doses of Eda treatment on MELAS ECs showed a dose-dependent decrease in the percentage of MitoSOX + cells (*p* < 0.001) (Fig. 6c, S5b). In addition to improving tube formation functions in MELAS ECs (Fig. 6d), Eda treatment also resulted in significant reduction in the expression of VCAM-1 isoform b in diseased ECs (Fig. S5c). Expectedly, this also led to a significant reduction in the number of monocytes adhering to the diseased ECs (~3-fold) (Fig. 6e). Taken together, anti-oxidant treatments on MELAS ECs were demonstrated to improve endothelial functions and in the process, lowering ROS levels and reducing the expression of VCAM-1 isoform b, thus bringing down basal inflammation level and reducing the activated status in MELAS ECs.

## Discussion

In recent years, MELAS has been reported to associate with the development of atherosclerotic lesions and cardiovascular diseases^10,31^. While the primary cause of SLEs in MELAS patients remains controversial, factors such as mitochondrial cytopathy, mitochondrial angiopathy and non-ischaemic neurovascular cellular mechanism, or combined, have been regarded as key contributing factors to disease onset and progression^32^.

Consistently, the bulk of our data validated earlier clinical reports that showed clear mitochondrial abnormalities in vessel biopsies of MELAS patients^33–35^. In addition, we also observed abnormally high basal level of endothelial inflammation in our MELAS EC vascular model. This was concluded through RNA-sequencing, increased IL-1β and IL-8 expression, and NF-_Κ_B translocation upon TNF*α* stimulation. The abnormally high basal inflammatory level also prompted us to investigate some of the inflammasome complex components in MELAS ECs and the results revealed upregulated expression of NLRP3, ASC and CASP1 (Fig. S4f, g) under both basal and stimulated (TNFα treatment) conditions. While the active form of caspase-1 was only found to be present in cMELAS EC upon TNFα treatment, MELAS ECs was found to express active caspase-1 and the expression level further increase upon TNFα treatment (Fig. S4g). Of note, the activation of pro-caspase-1 is required to process IL-1β precursor into its active, matured form that can then be secreted out of the cell to initiate the inflammation process^36^. Furthermore, VCAM-1 protein, in particular the isoform b, which has been postulated to be a pro-adhesive variation with enhanced interactions with inflammatory cells was observed to be significantly higher in the diseased ECs (Fig. 5c). This suggests the presence of a robust recruitment and interaction process whereby leucocytes and monocytes get adhered to the endothelial wall during inflammatory before infiltrating from the vessels to tissues^37^.

**Fig. 7:**
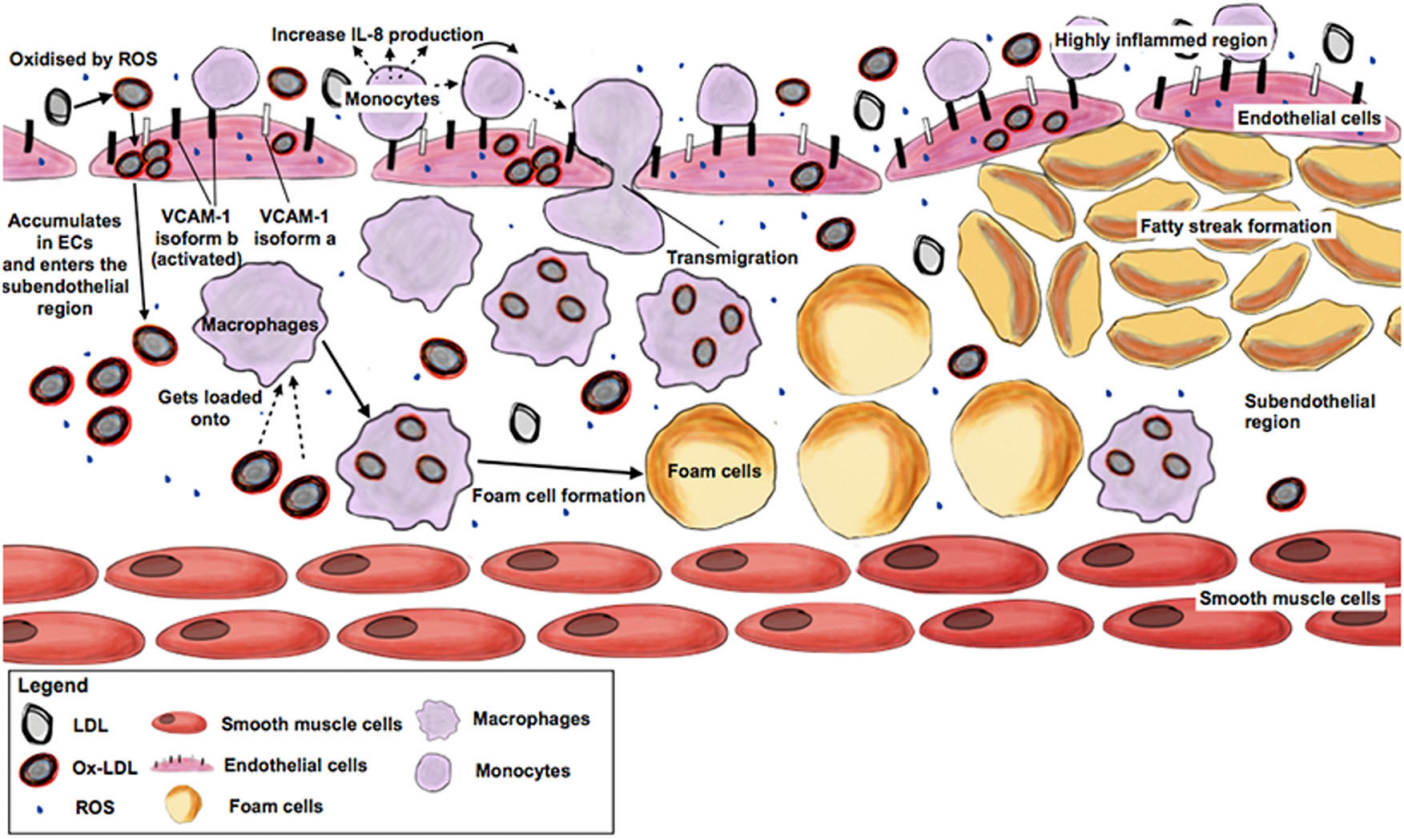
Schematic diagram summarising atherogenesis in the vasculature of MELAS patients. LDL gets oxidised to form ox-LDL by high levels of ROS in the environment. High levels of ox-LDL will be produced overtime and will accumulate in the ECs and subsequently, enters the sub-endothelial space. In tandem, monocytes in circulation attaches to activated ECs and become macrophages. High expression of ‘pro-adhesive’ VCAM-1 in these activated ECs promotes more monocytes to adhere to the endothelium. Large numbers of monocytes and macrophages creates a pro-inflammatory niche characterised by higher expression of chemokine IL-8. These macrophages then transmigrate into the sub-endothelial space where they are loaded with pathological levels of ox-LDL. Because of high lipid load, these macrophages transform into foam cells. Accumulation of foam cells in the vasculature overtime causes formation of the fatty streak and subsequently, an atherosclerosis plaque

Unsurprisingly, the mitochondrial ROS levels and expression of oxidative stress-related genes were found to be significantly higher in diseased endothelial cells as a result of complex I deficiency and disrupted electron transport chain activity due to the m.3243A > G mutation. Physiologically, ECs function to take up acetylated LDL circulating in the bloodstream via their scavenger receptors, a process critical for cholesterol metabolism, vascular inflammation processes and maintaining vessel tone^19^. In a ROS-rich environment such as those observed in MELAS ECs, circulating LDLs gets oxidised readily into ox-LDL^38^ (Fig. 4b). While Ox-LDL functions as a vasoconstrictor as well as a regulator of vascular inflammation at homeostatic conditions^39^, it contributes to endothelial dysfunction and atherosclerosis plaque formation at pathologically high levels. Underlying the high levels of modified LDLs seen in MELAS ECs is likely a result of poor degradation as expression of genes involved with modified LDL uptake appeared unchanged, while the expression of *LPL*, a key endothelial enzyme that hydrolyses lipoprotein triglycerides, was significantly reduced (Fig. S3a). Coupled with a high basal expression of VCAM-1 isoform b, our results provided evidence indicating a severe pro-atherogenic phenotype exhibited by MELAS ECs (summarised in Fig. 7), which suggests that MELAS patients suffer from a degree of atherosclerotic-like vascular vulnerability that significantly drive up their susceptibility to SLE development.

Oxidative stresses experienced by endothelial cells are a secondary effect of the energetic stresses present due to the defective electron transport chain. However, our approach has shown that the atherogenic risk presented in MELAS patients are primarily contributed to by ROS presence. As a proof of concept, treatment of MELAS ECs with anti-oxidants such as vitamin C and edaravone were found to successfully reduce expression of inflammasome components (ASC and CASP1) (Fig. S5b), and to restore tube formation capacity, in addition to reducing monocyte adhesion (Fig. 6). Of note, treatment with co-Q10 which merely restoring mitochondrial capacity was deemed insufficient, suggesting that the pathologically high levels of ROS in MELAS ECs must be addressed. Indeed, it is likely that current treatment options for MELAS involving l-arginine and citrulline supplementations, possess limited success because ROS levels in these patients remain pathologically high^32–35^. Edaravone, on the other hand, which is a more potent anti-oxidant, was previously shown to be useful in scavenging ROS and inhibiting inflammatory responses in the context of cerebrovascular diseases^40^. The usefulness of edaravone to improve vascular function of MELAS ECs was further demonstrated in the current study, suggesting the potential use of this compound as a treatment option for MELAS syndrome^41–43^.

In conclusion, we have found heightened levels of ROS and Ox-LDL in MELAS ECs to play critical roles in causing mitochondrial pathology. Coupled with high basal inflammatory level, as well as high VCAM-1 isoform b expression observed in the diseased ECs, a robust monocyte recruitment and adhesion process is present in MELAS patient, thereby promoting a pro-atherogenic phenotype. Henceforth, management of MELAS endothelial related pathologies could be administered at the level of oxidative stress inhibition as a treatment option for MELAS syndrome since it promotes endothelial cell survival and function, as well as preventing potential atherosclerosis plague formation.

## Materials and methods

### Cell culture and media

MELAS iPSCs were obtained through reprogramming of skin fibroblast of a male MELAS patient harbouring >80% of m.3243 A > G mutated mtDNA. MELAS iPSCs were subjected to genetic correction to completely remove the mutant mDNA as described by Yang et al. to obtain the MELAS isogenic control (cMELAS iPSC)^13^. The human H9 hES cell line and both iPS cell lines (MELAS, cMELAS) were cultured in feeder-free conditions on culture plates coated with Matrigel® matrix (Corning, U.S.A.) diluted in DMEM/F12 (Gibco, U.S.A.). Cells were maintained in StemMACS™ iPS-Brew XF basal medium supplemented with iPS-Brew XF, 50x supplement (Miltenyi Biotec, Germany) with 1% Pen/Strep (Gibco, U.S.A.) and were maintained in 37 °C under humidified atmosphere of 5% CO_2_. Culture medium was changed daily. After 80–90% confluency was reached, cells were passaged using 1 mg/ml Collagenase IV (Gibco, U.S.A.). When setting up the human pluripotent stem cells (hPSCs) for directed differentiation, the cells were passaged using StemPro-Accutase® as single cells (Gibco, U.S.A.) with 5 μM of Y27632 (Miltenyo Biotec, Germany). The U937 human monocytes (ATCC® CRL1593.2™) were cultured in RPMI media supplemented with 10% heat inactivated foetal bovine serum (FBS) (Hyclone, U.S.A.) and 1% Pen/Strep (Gibco, U.S.A.).

### Endothelial cells differentiation

Upon reaching 80–90% confluency, the human pluripotent stem cells (hPSCs) were induced to differentiate into mesodermal precursors for 36 h in a chemically defined medium as described by Narmada et al. with the addition of human recombinant fibroblast growth factor 2 (FGF2), LY294002 and human recombinant bone morphogenetic protein 4 (BMP4)^15^. For the subsequent 3.5 days, lateral plate mesoderm differentiation was further driven in medium supplemented with human recombinant FGF2 (20 ng/ml) and BMP4 (50 ng/ml), with a medium change every 2 days. On day 5, TrypLE Express was used to trypsinize the mesodermal population and then re-plated onto Matrigel-coated plates. The cells were then cultured in the same basal medium supplemented with FGF2 (4 ng/ml), SB431542, and vascular endothelial growth factor (VEGF) with medium change every 2–3 days. On day 10, endothelial cells expressing CD31 were sorted by using flurorescence-activated cell sorting. Approximately 5 × 10^5^ CD31 + cells were then plated onto rat tail Collagen-I (Corning, U.S.A.) coated well and cultured in Endothelial Growth Media-2 (EGM-2) (Lonza, U.S.A.) supplemented with 1uM SB431542 for 48 h before changing to fresh EGM-2 medium. Upon reaching 75–80% confluency, hPSC-ECs were passaged using TrypLE Express. EGM-2 medium was changed every 2–3 days. All cells used in the experiments do not exceed passage 10. For vitamin C (Vit C) treatment conditions, 2 dosages (100 μM and 500 μM) were selected and added into the EGM-2 medium and incubated for 24 and 48 h respectively. Similarly for Edaravone (Eda) treatment, cells were incubated in 100 μM, 200 μM and 500 μM of Eda for 24 and 48 h. Upon treatment, the cells were harvested for downstream assays.

### RNA extraction

For cultured cell samples, cells were collected and lysed in 300 μl of TRIzol reagent (Invitrogen, U.S.A.). The samples were allowed to stand for 5 min at room temperature, after which 180 μl of Chloroform (Kanto Chemical, Japan) was added to allow for phase separation by centrifugation at 12,000 × *g* for 15 min at 4 °C. Next, aqueous phase was transferred to a fresh tube with equal volumes of isopropanol and GlycoBlue Coprecipitant (Invitrogen, U.S.A.). The samples were incubated at room temperature for 20 min. The samples were pelleted through centrifugation at 12,000 × *g* for 15 min at 4 °C. The RNA pellet was washed with 100% ethanol, air-dried before reconstituting it in nuclease-free water (Ambion, U.S.A.).

### Reverse transcription and qPCR

RNA samples (500 ng) were reverse transcribed to obtain cDNA using High-Capacity cDNA Reverse Transcription Kit (Applied Biosystems, U.S.A.). qPCR was performed using the FAST SYBR Green Mix (Applied Biosystems, U.S.A.), 0.3 μM of specific primers (Supplementary Table 3) and ~5 ng of cDNA. ΔΔC_T_-based relative quantification method was adopted for qPCR analysis using the QuantStudio 5 384-well Block Real-Time PCR system (Applied Biosystems, U.S.A.). The threshold cycle was determined to be 40. Data is presented as fold-change where CT values were normalised to *β-ACTIN*. Data presented are representative of three independent experiments with error bars indicative of the standard deviation (s.d.) unless otherwise stated.

### Immunocytochemistry and confocal microscopy

Endothelial cells were dissociated to single cells for immunocytochemistry analysis through the use of TrypLE Express. Cells were then fixed using 4% PFA (Nacalai-Tesque, Japan) for 15 min at room temperature, followed by 0.2% Triton-X (Promega, U.S.A.) treatment for 15 min to permeabilise the cells when required. The cells were then treated with blocking buffer (PBS with 2% BSA and 5% Fetal Bovine Serum; Gibco, U.S.A.) for 30 min. Primary antibodies were diluted accordingly and incubated at 4 °C overnight. Cells were washed and then incubated with secondary antibodies conjugated with the appropriate Alexa Fluor® fluorescent dyes for 1.5 hours at 4 °C. Cells were then counter-stained with 4,6-diamindino-2-phenylindole (DAPI) (AAT Biorequest, U.S.A.) for nuclei visualisation. For this study, primary antibodies used are mouse anti-human CD31 (1:200), rabbit anti-human VCAM-1 (1:200), rabbit anti-human vWF (1:200), mouse anti-human eNOS (1:200), mouse anti-ATP5B (1:500), mouse anti-TOMM20 (1:500), rabbit anti-human Ki67 (1:1000), rabbit anti-human VeCAD (1:200) and mouse anti-human MTCO2 (1:200). Secondary antibodies conjugated with Alexa Fluor® 488 or 594 were then used for fluorescence detection. Cells were viewed and imaged using the Olympus Fluoview inverted confocal microscope (Olympus, U.S.A.).

### Flow cytometry analysis and fluorescence-activated cell sorting

ECs were first dissociated using Gibco® TrypLE Express (Thermo Fisher Scientific, U.S.A.) and centrifuged at 1200 rpm for 5 min. ECs cells were stained with antibodies that were diluted in blocking buffer in appropriate concentrations for 1–2 h at 37 °C and washed thoroughly with PBS before performing flow cytometry analysis using the LSR II (BD Biosciences, U.S.A.). For intracellular staining, there is an extra step of fixing cells with 4% PFA (Nacalai Tesque, Japan) at room temperature for 15 min before staining with antibodies. Cell pellet is finally resuspended in 500 μl–1 ml of fluorescence-activated cell sorting (FACs) buffer (PBS with 10% Knockout Serum Replacement; Gibco, U.S.A and 1% Pen/Strep; Gibco, U.S.A). For isolating CD31 + ECs, cells were stained accordingly as aforementioned and the cells were sorted using FACSAria II (BD Biosciences, U.S.A.) and collected in EGM-2 media supplemented with 1% Pen/Strep and 5uM of Y27632 (Miltenyi Biotec, Germany). Primary antibodies used include mouse anti- human CD31 Alexa Fluor® 647 (1:100) and rabbit anti-human VCAM-1 (1:500).

### Western blot

Cells were lysed using RIPA Buffer (ThermoScientific, U.S.A.) with the addition of cOmplete™ Protease Inhibitor Cocktail (Sigma Aldrich, U.S.A.). Protein extracts were then quantified using Pierce™ BCA Protein Assay Kit (Thermo Scientific, U.S.A.) in accordance to manufacturer’s recommendations. 4-20% SDS-PAGE Mini-PROTEAN® TGX Stain-Free gels (Bio-Rad, U.S.A.) were then used to separate proteins during electrophoresis. Subsequently, proteins were transferred onto 0.2 µM nitrocellulose membranes (Bio-Rad, U.S.A.) using the Trans-Blot® Turbo™ transfer system (Bio-Rad, U.S.A.). Primary antibodies used include rabbit anti-CASP7 (1:1000), mouse anti-ASC (1:100), rabbit anti-CASP1 (1:500), rabbit anti-human VCAM-1 (1:500) and mouse anti-human ATP5B (1:500). Secondary antibodies conjugated with horseradish peroxidase (Santa Cruz Biotechnology, U.S.A.) were used. Membranes were then developed using the Clarity™ ECL Western Substrate (Bio-rad, U.S.A.).

### Heteroplasmy determination by restriction fragment length polymorphism

Total genomic DNA were extracted from cells using the Nucleospin® Tissue kit (Macherey-Nagel, Germany). For RFLP analysis, the mtDNA m.3243 locus was amplified from the genomic DNA templates using primers mtDNA_3243 (Supplementary Table 3) using OneTaq® DNA polymerase (New England BioLabs, U.S.A.) to produce a PCR product of 634 bp^13^. In the presence of the m.3243 A > G mutation, an ApaI restriction site will be present. Digestion using ApaI restriction enzyme (New England BioLabs, U.S.A.) will result in two fragments - 424 and 210 bp. The digested PCR products were then separated on a 1% agarose gel using the FloroSafe DNA stain (1^st^ Base, Singapore). ImageJ was used to analyse the signal intensity of the bands.

### qPCR-based determination of relative mitochondrial DNA copy number

DNA samples were isolated using Nucleospin® Tissue kit (Macherey-Nagel, Germany). Next, 3 ng/μl of gDNA were used for qPCR as described by Rooney et al.^44^. Specific primers were designed for both mitochondria DNA and nuclear DNA (Supplementary Table 3). To determine mtDNA copy number, CT values of mitochondria DNA were normalised against nuclear DNA.

### Enzyme-linked immunosorbent assay

Human IL-8 ELISA kit (Invitrogen, U.S.A.) and ox-LDL ELISA kit (Cusabio Technology LLC, U.S.A.) were use to determine concentrations of IL-8 and ox-LDL respectively in cell lysate and conditional media samples in accordance to the manufacturer’s instructions.

### Tube formation assay

Matrigel® surfaces were prepared by the addition of 50 μl of neat Matrigel® (Corning, U.S.A.) into each well of a 96-well plate and made to solidify at 37 °C for 1 hour. 22.5 × 10^3^ of live ECs were seeded into each well. About 200 μl of EGM-2 medium was added into each well and cells were left to form tubes at 37 °C for 2–3 h. Brightfield images were taken using the Nikon Digital Sight DS-L3 camera (Nikon, Japan). Quantitative analysis of tube characteristics was performed by online software WimTube (Wimasis Image Analysis, Córdoba, Spain, http://www.wimasis.com).

### Acetylated low-density lipoprotein uptake assay

Cells were serum-starved for 1 h prior to incubation with 10 μg/ml of 3,3′-Dioctadecyloxacarbocyanine acetylated low-density lipoprotein (Ac-LDL) diluted in the EGM-2 media, at 37 °C with 5% CO_2_ for 4 h, unless otherwise stated. Live cells were then visualised and imaged using Olympus Fluoview FV3000 inverted confocal microscope (Olympus, U.S.A.).

### Apoptosis and cell viability analysis

To assess cell viability by flow cytometry, cells were stained with both DAPI (AAT Biorequest, U.S.A.) and Propidium Iodide (50 ug/ml) (Thermo Fisher Scientific, U.S.A.). For analysing apoptosis, cells were stained using the FITC Annexin-V apoptosis detection kit (BD Bioscience, U.S.A.) in accordance to manufacturer’s protocol.

### Scratch migration assay

A pipet tip was used to scratch a confluent monolayer of ECs in a straight line. Cells were washed once with PBS to remove debris and then replaced with fresh EGM-2 media. Afterwhich, the cells were incubated at 37 °C for 24 h and imaged to visualised the closure of the scratch. The acquired images were then imaged using online software WimScratch (Wimasis Image Analysis, Córdoba, Spain, http://www.wimasis.com).

### Mitochondrial superoxide detection

Cells were stained with 5uM MitoSOX™ Red reagent (Thermo Fisher Scientific, U.S.A.) in Hank’s buffered salt solution (HBBS) and incubated at 37 °C for 10 min. Cells were washed thrice with HBBS before analysis was performed via flow cytometry.

### Monocyte-adhesion assay

U937 human monocytes (ATCC® CRL1593.2™) were stained with Cell-tracker CM-Dil (Thermo Fisher Scientific, U.S.A.) according to manufacturer’s recommendation. About 5 × 10^5^ ECs were first seeded into a 12 well plate. Upon adhesion, 2 × 10^6^ of the previously stained monocytes were seeded onto the monolayer of ECs and incubate at 37 °C for 2 h before washing the non-adherent monocytes away thrice with PBS. Fresh monocyte culture media was added into the wells before imaging.

### Gene expression profiling and pathway enrichment analysis

The clean raw reads (fastq files) after adaptor removal with Trimmomatic^45^ were aligned to the reference human genome (hg19) by using STAR (v2.6.1a) with default parameters^46^. The gene expression quantification was done by using HTSeq-count (v0.9.1) in strand-specific mode to obtain raw read counts for each gene^47^. Then the differential gene expression analysis was performed by using DESeq2 starting from the raw read counts by comparing the samples from MELAS EC to cMELAS EC after collapsing the three samples within each group as replicates^48^. The genes that are not expressed (total read counts ≤1 across all the samples) were removed from the analysis. In total, 3503 and 3499 genes were significantly up- and downregulated, respectively, in MELAS EC compared to the cMELAS EC after correction for multiple hypothesis testing (*P*_adj_ < 0.05, using Benjamini-Hochberg method). Genes that were differentially expressed by $\ge 1.5$ fold change were selected for the gene ontology enrichment analysis based on the DAVID database^49^.

### Statistics

Quantitative PCR (qPCR) values are expressed as mean ± s.d. (unless otherwise stated) results were tested for statistical significance using Student’s *t*-test, two sided based on assumed normal distribution. Column statistics were performed using one-way ANOVA followed by Tukey post-hoc test. *p < 0.05, **p < 0.01 and ***p < 0.001 were considered to be statistically significant, very significant and highly significant, respectively.

## Supplementary information

Supplementary Information

## Accession code

RNA-Seq data have been deposited in GEO under accession ID GSE127478.
