# Supplementary material for: Mitochondrial 3243A > G mutation confers pro-atherogenic and pro-inflammatory properties in MELAS iPS derived endothelial cells
Source: Cell Death Dis. 2019 Oct 22;10(11):802. doi: 10.1038/s41419-019-2036-9 (PMC6805858; doi:10.1038/s41419-019-2036-9)
Supplement: Supplementary file 1 — Supplementary Information [file 41419_2019_2036_MOESM1_ESM.docx]

**Supplementary information**

**Supplementary table and table legends**

| **Up-regulated Pathway** | **Gene** |
| --- | --- |
| Inflammation | CFD |
|  | NPY5R |
|  | C3 |
|  | CFB |
|  | DHX58 |
|  | IL15 |
|  | IL18R1 |
|  | NPY5R |
|  | NUPR1 |
|  | P2RX1 |
|  | PDPN |
|  | STAT5A |
|  | TLR3 |
| Lipid transport and metabolism | ACADS |
|  | ACSL5 |
|  | AKR1C1 |
|  | AKR1C2 |
|  | APOC1 |
|  | CYP1A1 |
|  | HMGCS2 |
|  | OSBP2 |
|  | P2RX1 |
|  | PDPN |
|  | PIGL |
|  | PRLR |
|  | PTGES |
|  | STAT5A |
| cell adhesion | FREM1 |
|  | PRLR |
|  | PTK2B |
|  | STAT5A |
|  | TESK2 |
|  | VTN |

**Table 1.** List of up-regulated genes in MELAS vs isogenic control iPS derived endothelial cells (>2-fold). The genes were categorized into respective pathways.

| **Down-regulated Pathway** | **Gene** | |
| --- | --- | --- |
| Cell cycle | BIRC5 | KIFC1 |
|  | BLM | MCM10 |
|  | BRCA1 | NCAPG |
|  | BRCA2 | NCAPH |
|  | CDC25A | NDC80 |
|  | CDC45 | NUF2 |
|  | CDC6 | PBK |
|  | CDCA2 | PKMYT1 |
|  | CDCA3 | POLE2 |
|  | CDCA5 | POLQ |
|  | CDCA8 | PRUNE2 |
|  | CENPA | PSMC3IP |
|  | CHAF1B | PSRC1 |
|  | CLSPN | RAD51 |
|  | DSCC1 | RAD54L |
|  | DTL | RBL1 |
|  | E2F1 | RFC3 |
|  | ERCC6L | RNASEH2A |
|  | EREG | SESN3 |
|  | ESPL1 | SGOL1 |
|  | EXO1 | SGOL2 |
|  | FAM83D | SKA1 |
|  | GINS1 | SKA3 |
|  | GINS2 | SUV39H1 |
|  | GINS3 | TGFB2 |
|  | GINS4 | TIPIN |
|  | GSG2 | TK1 |
|  | GTSE1 | TRIP13 |
|  | HAUS8 | TUBB2B |
|  | HELLS | UHRF1 |
|  | KIF15 | ZWINT |

**Table 2.** List of down-regulated genes in MELAS vs isogenic control iPS derived endothelial cells (>2-fold). All the genes were found to be associated with cell cycle pathway.

| **Gene** | **Forward primer (5’ – 3’)** | **Reverse primer (5’ – 3’)** |
| --- | --- | --- |
| $\beta$*-ACTIN* | CCCATCGAGCATGGTATCATC | AGAAGCATACAGGGATAGCACT |
| *HMOX1* | GGCAGAGGGTGATAGAAGAGG | AGCTCCTGCAACTCCTCAAA |
| *NOX4* | GGGGTTAAACACCTCTGCCT | TCCTCGGAGGTAAGCCAAGA |
| *SOD1* | GCATCATCAATTTCGAGCAG | CAGGCCTTCAGTCAGTCCTT |
| *SOD2* | AATCAGGATCCACTGCAAGG | TAAGCGTGCTCCCACACAT |
| *MFN1* | GGGTGCTCCTAGGATTATCAGA | TATCTGGCGTTGCTGGAGT |
| *MFN 2* | TGCCTCAGAGCCCGAGTA | CTGGTACAACGCTCCATGTG |
| *MFF* | GGAGTTCCAAATGCTAGTGTGA | TGCTGGTCTTGAAAATGAAACA |
| *MIEF1* | CCACGCTGGCAGTTAAGC | CCCATCCAGTTGGGTTCTT |
| *MIEF2* | GACTTCCTCCTGGCCAATG | TGGCCCTGTCAATGAACC |
| *OPA1* | AACCATATTCGTTTTGACCAAAGT | TTGGGAAGAGCTTTCCTTCA |
| *FIS1* | CTGAACGAGCTGGTGTCTGT | AGCCTGCTGCCTTCTCAG |
| *ERRγ* | GCTGTTCTCCGCATCTATCC | TGAAATCACAAAGCGCAGAC |
| *PGC-1α* | CTGCTAGCAAGTTTGCCTCA | AGTGGTGCAGTGACCAATCA |
| *MT-ND1* | TCAAACTCAAACTACGCCCTG | GTTGTGATAAGGGTGGAGAGG |
| *MT-ND5* | CTACCTAAAACTCACAGCCCTC | GGGTAGAATCCGAGTATGTTGG |
| *NDUFA1* | TTCTAGCAGGGGTAGATGGC | GAGATAGGCGCATCTCTGGA |
| *SDHA* | CGAACGTCTTCAGGTGCTTT | AAGAACATCGGAACTGCGAC |
| *COX5B* | TCCATGGCATCTGGAGGT | TGTATGGGTCCAGTCCCTTCT |
| *ATP5B* | CAAGTCATCAGCAGGCACAT | GTGGGCTATCAGCCTACCCT |
| *PPARα* | GCTATCATTACGGAGTCCACG | TCGCACTTGTCATACACCAG |
| *PPARγ* | GAGCCCAAGTTTGAGTTTGC | GCAGGTTGTCTTGAATGTCTTC |
| *IL-6* | AAATTCGGTACATCCTCGACGG | GGAAGGTTCAGGTTGTTTTCTGC |
| *VCAM-1* | GGGAAGATGGTCGTGATCCTT | TGAGACGGAGTCACCAATCTG |
| *ICAM-1* | ACCGGAAGGTGTATGAACTGA | TGGTTGGCTATCTTCTTGCAC |
| *IL-8* | TTGGCAGCCTTCCTGATTTCTGCAG | ACAACCCTCTGCACCCAGTTTTC |
| *SDC1* | CTACAGCTGCCTCCACCTCC | CTGTGTGGTCTCCCTGGGTC |
| *NFKB* | ACTACCTGGTGCCTCTAGTGA | TTTGACCTGAGGGTAAGACTTCT |
| *L1CAM* | ACGAGGGATGGTGTCCACTTCAAA | TTATTGCTGGCAAAGCAGCGGTAG |
| *HLA-DPA1* | GCTTTGACCACTTGCATATTCAAACTGA | CCTTCCAGTTGGGCTACAGA |
| *ITGA2* | GAGCTTTTGTGATGGGCGATT | CTCTCCACCAACTTCATAAGGC |
| *OCT4* | TCAGCCAAACGACCATCTGCC | TTCTCTTTCGGGCCTGCACG |
| *SOX2* | ATGCCTTCATGGTGTGGTCCC | TCCGGGTGCTCCTTCATGTG |
| *NANOG* | AATGGTGTGACGCAGAAGGCC | TTGGAAGGTTCCCAGTCGGG |
| *T* | CTATTCTGACAACTCACCTGCAT | ACAGGCTGGGGTACTGACT |
| *MIXL* | GGCGTCAGAGTGGGAAATCC | GGCAGGCAGTTCACATCTACC |
| *EOMES* | GCCATGCTTAGTGACACCGA | GGACTGGAGGTAGTACCGC |
| *eNOS* | AAGGCTTTTGATCCCCGGGTCCT | TCTCCATCAGGGCAGCTGCAAAG |
| *vWF* | TAGCCCGCCTCCGCCAGAAT | CCTGCAGGCGCAGGTGAAGT |
| *CDH5* | TGGCCAGCTGGTCCTGCAGAT | TGCCCGTGCGACTTGGCATC |
| *CD31* | CAGGCGCCGGGAGAAGTGAC | CGTCCAGTCCGGCAGGCTCT |
| *LOX-1* | TGCCTGGGATTAGTAGTGACC | CCAGTTAAATGAGCCCGAGG |
| *LDLR* | CGTGCTTGTCTGTCACCTGCAAAT | AGAACTGAGGAATGCAGCGGTTGA |
| *CD36/ FAT* | AGATGCAGCCTCATTTCCAC | GCCTTGGATGGAAGAACAAA |
| *LRP1* | CTTTAATCGAGGGCAAAATGA | TGTCTTGGAGGTGACAAAGATG |
| *LPL* | GAGTAGCAGAGTCCGTGGCT | CGCGGACACTGGGTAATGCT |
| *EL* | TGGATCTTTCGGACTGAGGCCT | ATGCAGGCCAATGGTAGGGACT |
| *PYCARD* | AACCCAAGCAAGATGCGGAAG | TTAGGGCCTGGAGGAGCAAG |
| *NLRP3* | GATCTTCGCTGCGATCAACA | GGGATTCGAAACACGTGCATTA |
| *CASP1* | GCCTGTTCCTGTGATGTGGAG | TGCCCACAGACATTCATACAGTTTC |
| *IL- 1β* | CCAGGGACAGGATATGGAGCA | TTCAACACGCAGGACAGGTACAG |
| mtDNA 3243 locus | CCTCGGAGCAGAACCCAACCT | CGAAGGGTTGTAGTAGCCCGT |
| tRNA-Leu (UUR) | CACCCAAGAACAGGGTTTGT | TGGCCATGGGTATGTTGTTA |
| B2-microglobulin | TGTTGTCTCCATGTTTGATGTATC | TCTCTGCTCCCCACCTCTAAGT |

**Table 3.** List of qPCR primers used in this study.

**Supplementary figures and figure legends**

**
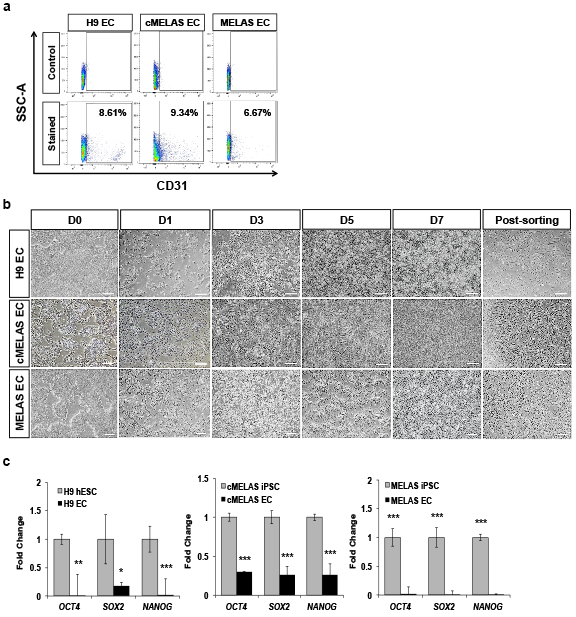
**

**Figure S1. a)** Flow analysis of CD31 showed significantly lower CD31^+^ ECs after MELAS iPSCs were differentiated into ECs. **b)** Brightfield images showing morphological changes undergone by hPSCs during EC differentiation. Scale bar = 100$\mu$m. **c)** Transcriptome profile of pluripotency genes (*NANOG, SOX2* and *OCT4*) showed successful EC differentiation; ECs derived from the hPSCs expressed significantly lower levels of pluripotent genes as compared to the respective hPSCs. Results are represented as fold change normalised to $\beta$-*ACTIN*. Error bars show SD of the mean. *p < 0.05, **p < 0.01, ***p < 0.001.

**
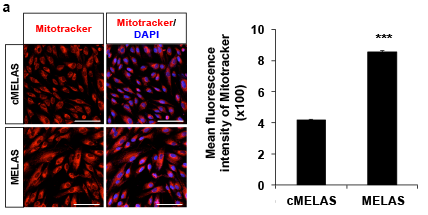
**

**Figure S2. a)** Representative images of live ECs stained with Mitotracker Red reagent showing MELAS ECs had higher mean fluorescence intensity of Mitotracker as compared to the isogenic control (n>100 cells). Nuclei were stained in blue with DAPI. Scale bar = 100$\mu$m. Error bars show SD of the mean. ***p < 0.001.

**
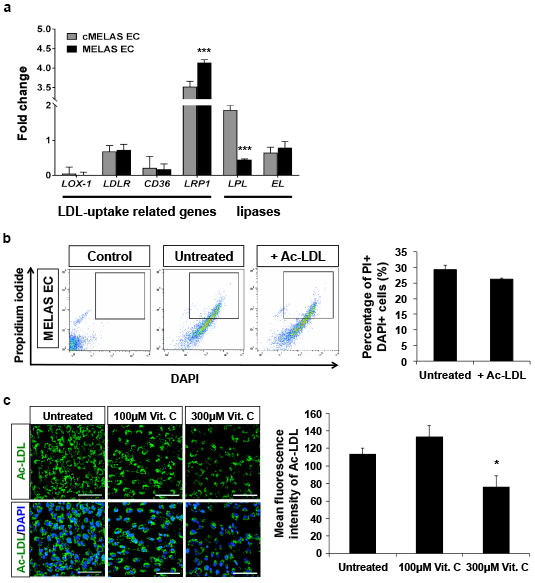
**

**Figure S3. a)** Gene expression of several LDL uptake-related genes and lipases. Expression of *LRP1* was significantly upregulated in MELAS ECs while expression of *LPL* was observed to be downregulated in MELAS ECs. Data are represented as fold change normalised to *β-ACTIN*. **b)** Flow cytometric analysis of PI and DAPI in live ECs showed that there is no significant cellular death (PI+/DAPI+ cells) after Ac-LDL treatment. **c)** Representative images of Ac-LDL in ECs after treatment with 100$\mu$M and 300$\mu$M of Vit C. Mean fluorescence intensity of Ac-LDL was significantly reduced when MELAS ECs were treated with 300$\mu$M of Vit. C. Nuclei were stained in blue with DAPI. Scale bar = 100$\mu$m. Error bars show SD of the mean. *p < 0.05.

**
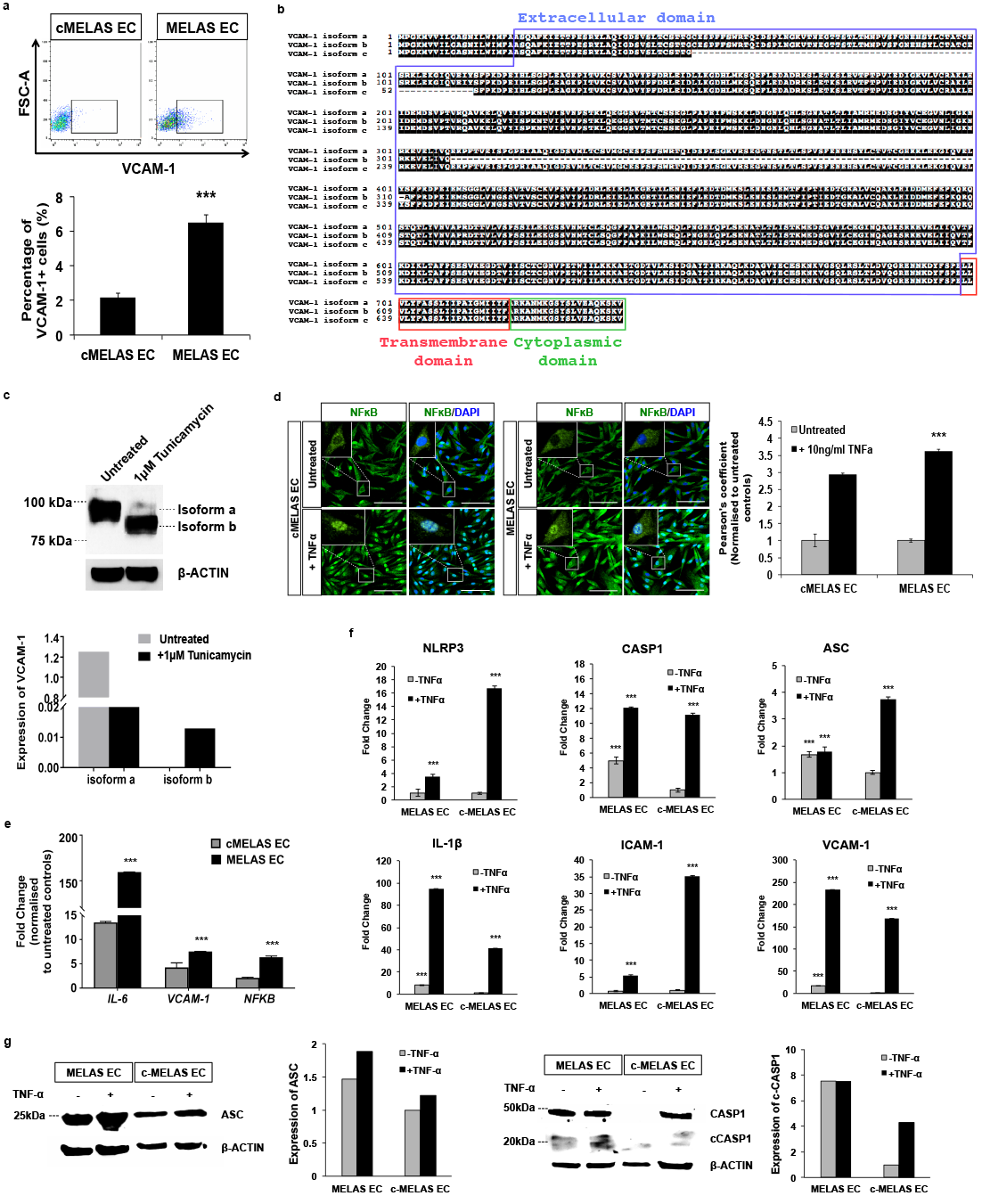
**

**Figure S4. a)** Flow cytometric analysis based on VCAM-1 expression revealed higher percentages of VCAM-1+ ECs in MELAS as compared to the isogenic control. **b)** Multiple-sequence alignment showed that the known VCAM-1 isoforms differ mainly in the amino acid sequence of the extracellular domain (boxed in blue). **c)** cMELAS ECs expressed significantly more VCAM-1 isoform b upon treatment with 1$\mu$M of Tunicamycin. Expression of VCAM-1 isoforms were quantified and normalized to loading control $\beta$-ACTIN. **d)** Representative Immunostaining images of NF-_Κ_B p65 (RelA) in cMELAS and MELAS ECs. Nuclei were stained in blue with DAPI. Scale bar = 100$\mu$m. Quantification of nuclear co-localization of NF-_Κ_B levels was performed and represented by mean Pearson’s coefficient ± s.d. **e)** Expression of NF-_Κ_B was upregulated upon TNFα treatment. **f)** Gene expression of several inflammasome related genes (*NLRP3*, *ASC* and *CASP1*) and adhesion markers (*ICAM-1* and *VCAM-1*). **g)** MELAS ECs expressed significantly higher basal levels of inflammasome related proteins, ASC and CASP1. Further upregulation of these proteins were observed in MELAS and cMELAS ECs upon TNFα treatment. Error bars show SD of the mean. ***p < 0.001.

**
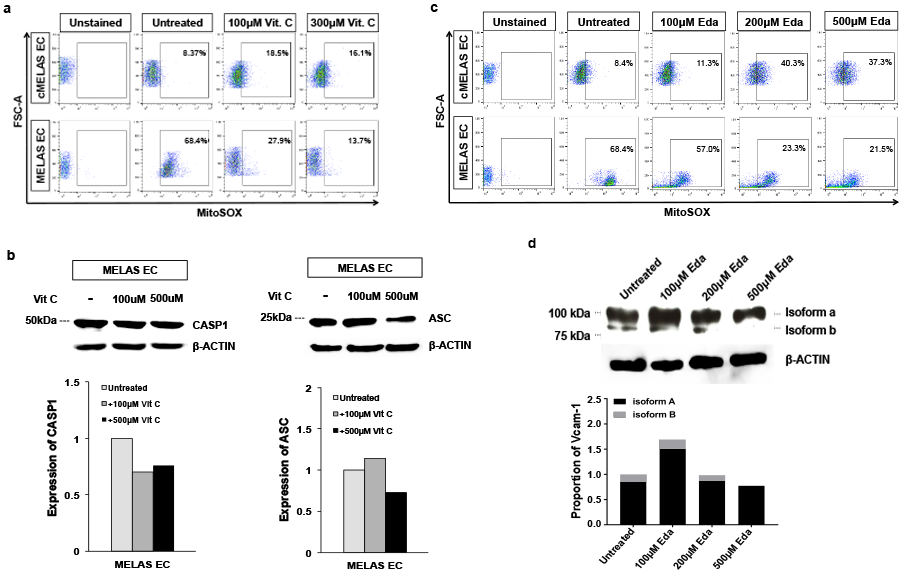
**

**Figure S5. a)** Flow cytometric plots showing Vit. C treatment was effective in reducing the number of MitoSOX^+^ cells in MELAS ECs. **b)** Western blot and densitometric analysis showing increasing doses of vitamin C resulted in the reduction of inflammasome associated proteins (CASP1 and ASC) expressions in a dose-dependent manner. **c)** Flow cytometric plots showing increasing doses of edaravone was effective in reducing the number of MitoSOX^+^ cells in MELAS ECs in a dose-dependent manner. **d)** Western blot and densitometric analysis shows increasing doses of edaravone successfully reduced the expression of VCAM-1 isoform b in a dose-dependent manner.
